# Palikur traditional roundwood construction in eastern French Guiana: ethnobotanical and cultural perspectives

**DOI:** 10.1186/s13002-018-0226-7

**Published:** 2018-04-24

**Authors:** Clémence Ogeron, Guillaume Odonne, Antonia Cristinoi, Julien Engel, Pierre Grenand, Jacques Beauchêne, Bruno Clair, Damien Davy

**Affiliations:** 1 LEEISA (Laboratoire Ecologie, Evolution, Interactions des Systèmes Amazoniens), CNRS, Université de Guyane, IFREMER, 275 route de Montabo, 97300 Cayenne, France; 2 CNRS, UMR Ecologie des Forêts de Guyane (EcoFoG), AgroParisTech, Cirad, INRA, Université des Antilles, Université de Guyane, 97310 Kourou, France; 3 LLL, Laboratoire Ligérien de Linguistique, UMR 7270, Université d’Orléans, 45065 Orléans, France; 4 ICTB (International Center for Tropical Botany, Department of Biological Sciences), Florida International University, Miami, FL 33199 USA; 5 AMAP, IRD, CIRAD, CNRS, Université de Montpellier, INRA, Boulevard de la Lironde, TA A-51/PS2, F-34398, Montpellier Cedex 5, France; 6 CIRAD, UMR Ecologie des Forêts de Guyane (EcoFoG), AgroParisTech, Cirad, INRA, Université des Antilles, Université de Guyane, 97310 Kourou, France

**Keywords:** Oyapock, Palikur, Traditional technological knowledge, Amazonia, Annonaceae, Sapotaceae, Non-timber forest products, Architecture

## Abstract

**Background:**

Palikur Amerindians live in the eastern part of French Guiana which is undergoing deep-seated changes due to the geographical and economic opening of the region. So far, Palikur’s traditional ecological knowledge is poorly documented, apart from medicinal plants. The aim of this study was to document ethnobotanical practices related to traditional construction in the region.

**Methods:**

A combination of qualitative and quantitative methods was used. Thirty-nine Palikur men were interviewed in three localities (Saint-Georges de l’Oyapock, Regina and Trois-Palétuviers) between December 2013 and July 2014. Twenty-four inventories of wood species used in traditional buildings were conducted in the villages, as well as ethnobotanical walks in the neighboring forests, to complete data about usable species and to determine Linnaean names.

**Results:**

After an ethnographic description of roundwood Palikur habitat, the in situ wood selection process of Palikur is precisely described.

A total of 960 roundwood pieces were inventoried in situ according to Palikur taxonomy, of which 860 were beams and rafters, and 100 posts in 20 permanent and 4 temporary buildings. Twenty-seven folk species were identified. Sixty-three folk species used in construction were recorded during ethnobotanical walks. They correspond to 263 botanical species belonging to 25 families.

Posts in permanent buildings were made of *yawu* (*Minquartia guianensis*) (51%) and *wakap* (*Vouacapoua americana*) (14%). Beams and rafters were made of wood from Annonaceae (79%) and Lecythidaceae (13%) families. The most frequently used species were *kuukumwi priye* (*Oxandra asbeckii*), *kuukumwi seyne* (*Pseudoxandra cuspidata*), and *pukuu* (*Xylopia nitida* and *X. cayennensis*).

**Conclusions:**

Although the Palikur’s relationship with their habitat is undergoing significant changes, knowledge about construction wood is still very much alive in the Oyapock basin. Many people continue to construct traditional buildings alongside modern houses, using a wide array of species described here for the first time, along with the techniques used.

**Electronic supplementary material:**

The online version of this article (10.1186/s13002-018-0226-7) contains supplementary material, which is available to authorized users.

## Background

Numerous ethnobotanical works highlight the richness of ethnobotanical and ethno-ecological knowledge of Amerindian people. In French Guiana, this is particularly true of the Wayãpi Amerindians [29, 30]. Most of these works focus on medicinal plants [33, 47] and some advanced studies on technical plants used for crafts, e.g., basketry [15, 16], bows, and arrows [31], or for dyeing [34]. Conversely, the diversity of woody species used as building material for housing remains insufficiently explored [21, 30] even though the architectural relevance and perfect adaptation of the Guianan habitat to its environment has been demonstrated in several cultural groups [1, 10, 38, 54].

At the Amazonian level, roundwood is one of the most important non-commercial logging forest products still used today for subsistence by many forest-dwelling people [18, 61]. Several ethnobotanical works claim that the wood of a wide range of species is suitable for construction, including all forms of permanent or temporary dwellings or any other structures (primarily small-diameter roundwood used for posts and beams and sometimes sawn boards used for walls or for canoes) [55, 65]. This obviously excludes any commercial timbers collected on an industrial scale. Indeed, depending on the community and location, the number of recorded species used for construction (with a diameter > 10 cm) ranges from 3% of total woody species (Panare in Venezuela) to 56% (Arawak in Guyana) [4, 5, 8, 11, 12, 17, 30, 44, 51, 53, 55, 56, 58, 63, 65]. Some authors show that the choice of the species depends on the planned use, i.e., either as posts or for the framework, as these uses do not require the same degree of durability [5, 11, 17, 30, 42, 44, 52, 55, 56, 65]. Despite this assessment, only one ethnobotanical study specifically focused on the use of wood in housing, combining an ethnographic description and a quantitative approach to the diversity of woody species used in a traditional Yanomami housing structure in Brazil [42]. The material, architectural, and, in some anthropological studies, symbolic dimensions of Amazonian habitats have been the main focus to date [3, 20, 24, 25, 36]. According to these references, and even if architectural typology and techniques are specific to each community, the general pattern of Amerindian construction in Amazonia (sensu *lato*, including in the Guiana shield) appears to be based on an erected roundwood framework, lashed together and thatched with palms [5, 19, 20, 24, 25, 36, 38,42, 44, 54, 56, 65].

The Palikur people (*Pahikwene*) speak an Arawak language (*pahikwaki*) and originate from the northeast of Amapá (Brazil). The Palikur have been present in Amapá and the Oyapock region since the sixteenth century [32]. They now live in a region stretching from the central part of the coast of French Guiana to the extreme northeast of Amapá (Brazil). The Brazilian and French Palikur still maintain regular matrimonial and cultural exchanges. In 2001, the total population was approximately 1800 people, of whom approximately 850 live in French Guiana [27]. A Palikur community has long been established in the district of Saint-Georges de l’Oyapock, including Trois-Palétuviers village since 1960, and their population is now estimated at around 500 people among a total population of approximately 2000 Palikur.

Recently, the lower Oyapock basin has undergone significant changes, especially since the construction of a national road in 2003, and changes will certainly be intensified as the bridge between French Guiana and Brazil opened [13, 28]. Despite major changes in habitat with the arrival of new building materials and a very old Western influence that dates back to the sixteenth century, the Palikur community is the only one in this area to conserve a traditional architectural typology as described by Mattioni [40], Nimuendaju [46], and Pérez [50]. Major technological and cultural shifts, evangelical influence, added to the process of assimilation in the 1960s, when traditional practices and ceremonial life were discouraged, led to deep modifications in their housing habits [27]. Like many other Amazonian peoples, the Palikur abandoned their former communitarian and semi-permanent housing for individual stilt houses based on neo-Amazonian models, which led to a new social and cultural order. Their settlement close to administrative centers also influenced their socio-cultural habits. Lastly, these transformations are related to several social housing programs that began in the early 1990s, when the Palikur slowly transformed their houses to resemble Western buildings [10]. Roundwood buildings have thus tended to lose their housing function, which is now more associated with concrete private houses. Traditional buildings are more and more limited to the role of annex buildings, dedicated to collective and communitarian life, or to daily tasks.

Due to the scarcity of data on traditional construction in north-eastern Amazonia and of in-depth studies on this topic and to prevent the loss of knowledge or its transformation due to the context of change, publishing the data gathered in the present study is important for both the Palikur and the scientific community.

To understand how the Palikur build traditional houses and which species they use, we (1) provide an ethnographic description of Palikur roundwood habitat, nomenclature, and techniques; (2) identify and quantify the diversity of woody species used for construction; and (3) compare our data with other Amazonian ethnobotanical studies.

## Methods

### Biodiversity and access to traditional knowledge

Traditional Palikur chiefs in the district of Saint-Georges de l’Oyapock were consulted at the beginning of the study and gave their verbal consent for our work. The chiefs were regularly informed about the progress of the project and were again consulted in September 2017 to obtain their agreement for publication of the results. Herbarium vouchers were collected in the national forest of “Régina-Saint-Georges de l’Oyapock” with the authorization of the French National Forestry Office (ONF) (no. 140523_02/MH/ML).

### Study site

**Fig. 1:**
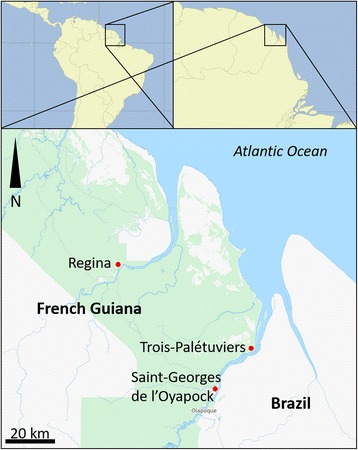
Location of the study area

The survey was conducted in the district of Saint-Georges de l’Oyapock, from December to July 2014, in the village of Trois-Palétuviers in November, and in the village of Régina from April to May 2014 (Fig. 1). The forest cover area is quite homogeneous and mainly of the *terra firme* type, growing on crystalline rocky substrate, with predominance of species belonging to the Leguminosae (Caesalpinioideae) family [7, 35]. The climate is wet equatorial, and the average yearly rainfall is 3500 mm, with a rainy season (December to July) and a dry season (August to November) [7].

### Open ended interviews

In Palikur culture, construction is considered a male activity [46]. This gendered distribution led us to conduct a first set of open-ended interviews with Palikur men of an age to build their own houses. The interviews were conducted in the Palikur language (*pahikwaki*) if the interviewee so desired, otherwise in French.

### Structure nomenclature and techniques

After identifying reliable informants in preliminary open-ended interviews, each category of wood components of each roundwood building was numbered and the Palikur names were recorded. Detailed drawings were used to facilitate dialog with informants. Each informant was interviewed separately to limit the influence of the other people present. Additional data such as the description of construction techniques and nomenclature were collected in semi-directed interviews. All the Palikur vocabulary was cross-checked in a final field campaign in January 2016.

### Inventories of construction wood

Systematic quantitative surveys of the names of the wood used for each part of the frame were conducted using 24 constructions (14 in Saint-Georges de l’Oyapock, 8 in Trois-Palétuviers, and 2 in Régina) with the builders. Folk names in Palikur were used during this part of the survey. Assembly techniques, thatch materials, and the description of the wood were also recorded.

### Ethnobotanical walks

Ethnobotanical walks were undertaken in the surrounding forest with 13 Palikur men to identify the species that corresponded to each folk name of wood previously identified as being used in the building. The men were paid as guides for this part of the work. The names of the species of wood names in Palikur and their uses were cross-checked to insure consensus. Herbarium vouchers, photographs, and GPS coordinates were collected for each tree.

### Plant collection and identification

Voucher specimens were immediately preserved in 50% ethanol until processing and incorporation in the herbarium of the “Institut de Recherche pour le Development” (IRD) in Cayenne, French Guiana (CAY). Identifications were made by C. Ogéron, J. Engel, and G. Odonne, assisted by professional botanists P. Delprete (Rubiaceae), R. Girault (generalist), E. Lucas (Myrtaceae), P. Maas (Annonaceae), and P. Petronelli (Guiana tree generalist). The taxonomic validity of species, genera, and families was checked according to APG IV [2] via the Taxonomic Name Resolution Service [14]. The supplementary ethnobotanical material (herbariums, folk names) was obtained from the CAY herbarium. These unpublished data had been collected in previous general Palikur ethnobotanical surveys conducted between 1978 and 2015. This involved a review of 261 herbarium vouchers deposited in the Cayenne herbarium and collected by P. Grenand, M. F. Prévost, G. Cremers, C. Moretti, H. Jacquemin, J. P. Lescure, M. E. Berton, D. Davy, G. Bourdy, J. J. Piolat, and S. Rostain (order of decreasing importance). One hundred seventy-five trees identified by J. Engel and P. Petronelli and named by one of the informants in a permanent forest plot located between Saint-Georges de l’Oyapock and Regina in a previous research project were added to the vouchers. These supplementary data were used to improve consensus between folk and botanical taxonomy.

### Data analysis

A fidelity index (FI) was defined for each species of wood cited to quantify inventory data. The index was adapted from Friedman et al. [23] as follows: Fl = Np/N (where Np is the number of informants who independently claim a specific use of a wood in a building or in the forest and N is the total number of informants who use this wood for any use, to quantify the use consensus between all informants [41]).

## Results

### Ethnographic description and typology of roundwood Palikur habitat

Thirty-nine men were interviewed (21 from Saint-Georges de l’Oyapock, 13 from Trois-Palétuviers, and 5 from Régina), representing approximately 20% of the adult Palikur male population of north-eastern French Guiana. The average age of the surveyed population was 50 (ages ranged from 20 to 77).

According to the informants, Palikur used to live in circular collective houses (*payt masikahaki*) covered by a conical roof, thatched with palms and extending to the ground, as also reported by Mattioni [40] and Pérez [50]. The structure of the roof recalls that of the nest of the *motye masik* wasp, *Apoica pallida* (Vespidae). However, archives of the seventeenth and eighteenth centuries only evoke rectangular collective houses on stilts. The first architectural type has now disappeared and has been replaced by rectangular, open buildings, topped by a ridge. These buildings have a wooden framework made of small diameter roundwood, close to the generic type of housing in Amazonia. When the Palikur from Saint-Georges de l’Oyapock occupied the inundated savannas and forested islands of the Arucauá in Brazil, they built houses on stilts. These had a raised floor (*payt avaakap*) made of ax-split palm tree stipes or of wooden planks (*paak*) cut with European saws [40, 46]. A trunk with carved steps (*araybu*) was used as a ladder to access the living area [40]. Today, this kind of housing is only found in small settlements located near rivers and marshes in Brazil. Other houses that were erected on firm ground had a dirt floor. The second type is the main form found in eastern French Guiana today. Since Palikur settled permanently in Saint-Georges de l’Oyapock in the 1960s, housing has been continually changing, notably due to relocation programs that started in the 1990s. Traditional housing is progressively being replaced by contemporary, individual, concrete houses (*payt bataka*). These are prefabricated, concrete, one-story houses, sometimes closed with wood cladding, covered with a corrugated iron roof (*avinvit sivariptiye*). The last houses (*payt himeket*) that still have a raised floor are closed individual ones. They are built on stilts, and the style is between that of traditional Palikur roundwood houses and Amazonian *cabocla* houses [50]. The frame still has the traditional appearance, but the round posts have been replaced by sawn wood. Despite these changes, roundwood housing is still definitely present.

**Fig. 2:**
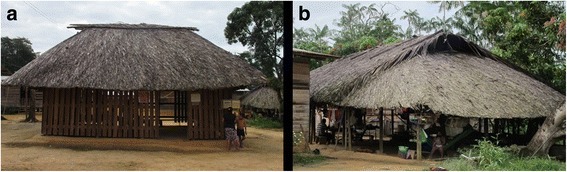
Left, **a** ceremonial building with a simple hip roof. Right, **b** simple hip building with apses, used for cassava flour preparation

Permanent roundwood Palikur housing groups four distinct functional types, of which two are still dedicated to community life (Table 1). One of these buildings is where meetings, ceremonies, councils, and cultural activities take place (*payt pahadrunket*, Fig. 2a). The other is used by the families to make roasted cassava flour (*payt ihevinwa*, Fig. 2b). Although these buildings are sometimes located in the plantations themselves, nowadays they are usually associated with the home. Their architecture is traditional with a simple hip roof, with or without apses, including triangular vent holes (*miokye*) both to allow the smoke to escape and to ventilate the structure.

**Table 1:** Palikur habitat names and their meaning

Palikur name	Etymology	English name	Type
*Payt hihevinwa* (syn.: *payt hihe avin*)	*Payt*: house *Hihe*: pan	Cassava house	Permanent
*Payt pahadrunket* (syn.: *payt kayket*)	*Payt*: house *Pahadrunka*: meeting *Kayka*: to dance	Meeting house	Permanent
*Payt himeket*	*Payt*: house *Himekne*: to sleep	Sleeping house	Permanent
*Payt sakehweket* (syn.: *payt tevweyeket)*	*Payt*: house *Sakehwene*: to cook, to boil *Tevwene*: to roast	Cooking house Roasting house	Permanent
*Payt pawkavinwa*	*Payt*: house *Pawka*: far	Plantation house	Temporary
*Payt timuvugaib*	*Payt*: house *Timuvu*: hocco (bird) *Crax alector* *Gayb*: tail	Hocco’s tail shelter (small hunting shelter)	Temporary
*Payt wewvaki*	*Payt*: house *Wewvene*: to hunt	Hunting shelter	Temporary
*Pew gavin*	*Pew*: dog *-Vin*: house	Kennel	Temporary
*Takaak gavin*	*Takaak*: chicken *-Vin*: house	Aviary	Temporary

*Syn.* synonym

**Fig. 3:**
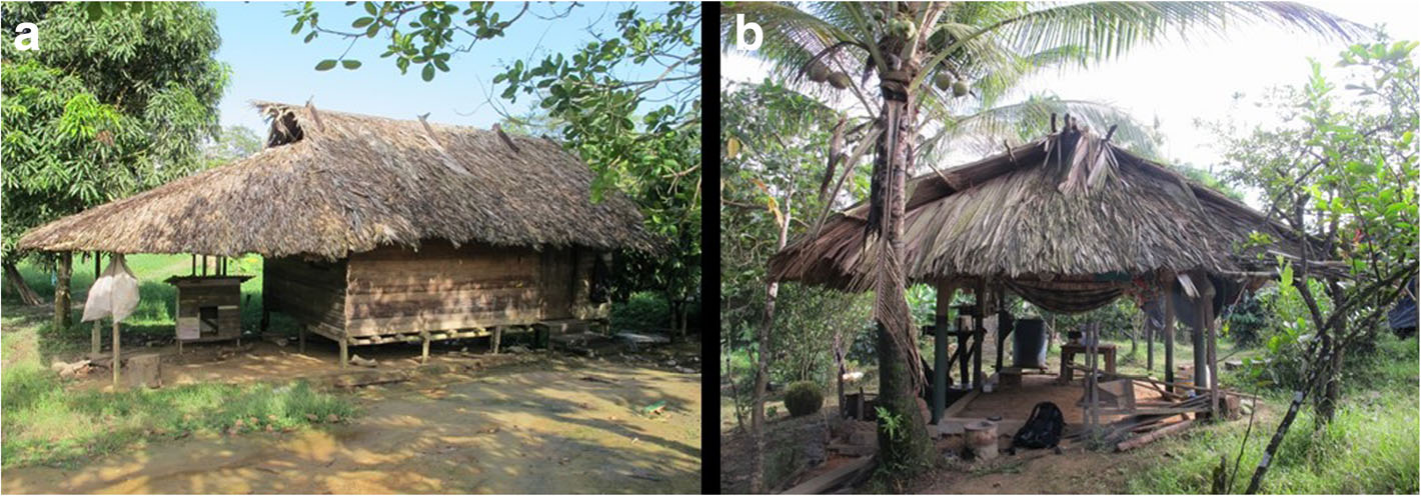
Left, **a** living house on stilts. Right, **b** cooking and resting building annexed to a house

In addition to these buildings, there are individual buildings with gable roofs, including a handful of homes (*payt himeket*) built on stilts (Fig. 3a), whose flooring is at a height of 80 cm above the ground. They are sealed with wood cladding (*pitimka*) obtained from industrial sawmills in the neighboring Brazilian city of Oiapoque. One of the gables is often extended to form a lean-to (*payt adaka*), added as protection against the prevailing wind [40]. A small cooking (*payt sakehweket*) or meat grilling (*payt tevweyeket*) hut is often added and is sometimes used as a resting place (Fig. 3b).

**Fig. 4:**
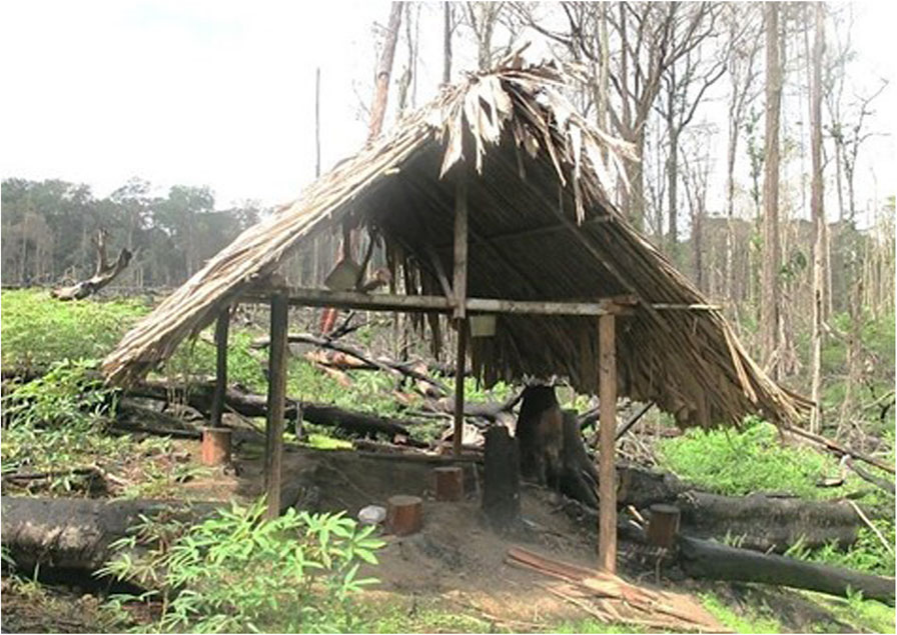
Light shelter in a cassava field

Gable roof shelters found in plantations (*payt pawkavinwa*) are built to last for short periods of time (Fig. 4), and their structure is lighter. Temporary buildings also include individual shelters used on hunting trips (*payt timuvugaib*, meaning literally *Crax alector* bird tail like shelter), as well as bigger ones used as collective housing during hunting trips (*payt wewvaki*). Aviaries (*takaak gavin*) and kennels (*peu gavin*) are also light buildings.

### Construction of Palikur roundwood permanent buildings

All the materials (wood, bonds, palms) used in a roundwood building are of plant origin and are harvested in the neighboring forest.

#### In situ tree selection, recognition, and harvest

The recognition and choice of trees in the forest is a progressive process, involving several cognitive criteria. The first criterion depends on the selected use. For posts, as the number of species used is small, the selection is first based on identification of the species, then on the rectitude and the diameter of the trunk (> 20 cm). Concerning roundwood for the frame, the first criterion is the trunk geometry. Palikur prefer small-diameter trees (around 15 cm maximum), with both a straight and cylindrical trunk (as opposed to angular, buttressed trunks). The hardness of the wood is first estimated by the sound produced when the trunk is hit with a machete. Hardwoods are most prized and are recognizable by the clear sound they emit and by the fact that they offer greater resistance to the machete’s blade. Species are also often identified by making a slash in the bark and trunk wood with a machete. Identification involves observation of various phenotypic traits: trunk shape, wood and bark color, texture, smell, taste, and presence of exudates.

Trees are preferably felled in the waning moon phase, which is thought to prevent infestation by xylophagous insects. The most frequently cited woodborers are termites (*mun*) (genera *Heterotermes*, *Coptotermes*, *Nasutitermes*) or wood-eating beetles (*uvis*) (genera *Lyctus*, *Bostryches*, *Anobium*, *Scolytes*). Wood cut during this period is also thought to be less likely to split. Trees are cut down with axes or machetes, but chainsaws are progressively replacing them.

#### Wood transformation process

The straight cylindrical part of the trunk, measuring from 10 to 15 m, is prepared in situ. The branches are removed with a machete, and then, the bark is stripped from the boles. A machete is used for Annonaceae and Lecythidaceae whose barks are easy to strip off. The blade is inserted along the cambium between the wood and the bark. For the Chrysobalanaceae, which have a more adhesive bark, the bole is hit with a large stick and scratched with the blade of the machete to peel off the bark. The aim of these processes is to prevent attacks by wood-eating pests and to allow the wood to dry and harden more quickly. In temporary buildings, the bark is sometimes not stripped off, as it peels off spontaneously after drying. The boles are then carried by hand to the construction site, with the assistance of a motor vehicle if available. They are then cut into pieces of the appropriate length for the desired building. These steps are always carried out on green wood, as it hardens and deforms while drying. Depending on the species used, the pieces destined to be used as posts may have their sapwood (*ã gasisin*) (peripheral, non-durable wood) removed with an ax, conserving only the heartwood (*ã gayakni*). This finishing step was observed only once and aims to prevent the wood rotting and to avoid insect attacks.

#### Material processing, assembly techniques, and structure nomenclature

**Fig. 5:**
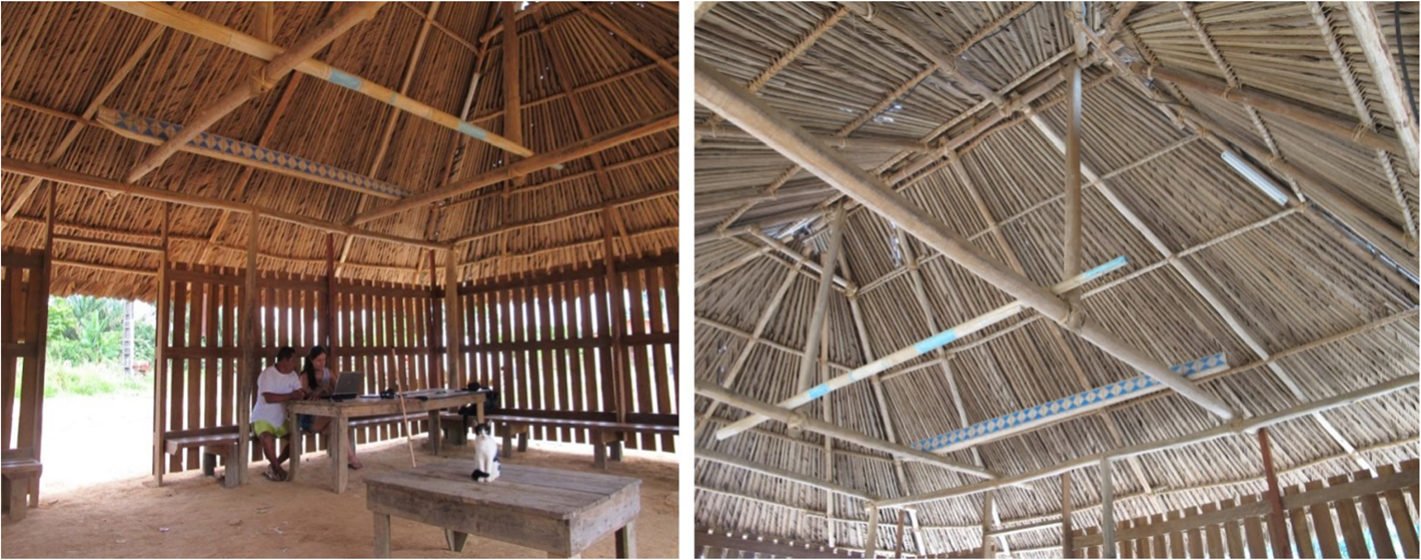
Ceremonial building at Esperance village, from the inside (same building as in Fig. 2a)

**Fig. 6:**
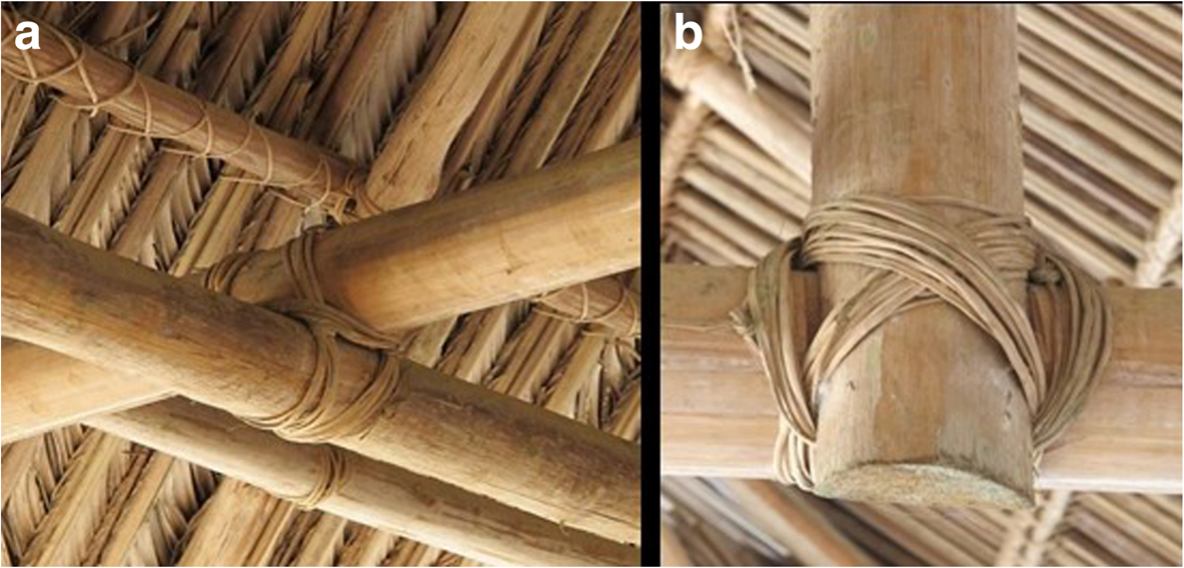
Lashings (*wanaka*) with *M. saccifera* palm cover in the background. Left, **a** square lashings. Right, **b** diagonal lashings

Once the location of the building has been chosen, the first step is setting up the vertical frame (Fig. 5). The length between two posts is measured in fathoms, i.e., the space between the two hands, arms open wide. Posts (10–15 cm in diameter) are buried to a depth of 50–80 cm in holes in the earth floor. Girders are placed on top of the posts in mid-wood notches (*araybu*). The structural elements that rely on these are tied together and secured by square or diagonal lashing (Fig. 6). Their names in Palikur are listed in Table 2.

**Table 2:** Palikur names of structural elements and their English equivalent

Structure element	Palikur name
Post	*Wakap*
Bridging beam	*Ayabwi dahagbukya wakap*
Tie beam	*Adaap*
Crown post or king post	*Akoksa*
Lower ridge piece	*Ayabwi dahagbukya akoksa*
Upper ridge piece	*Ayabwi avugekuya*
Common rafter	*Avayta*
Roof rafter	*Avayta amagtongitak*
Angle rafter	*Avayta savogene*
Upper tie beam	*Atawkan adah kamaxne avayta amagtõgitak*
Batten of roofing	*Akanwapti*
Wind brace	*Asugetni*
Hipped roof	*Payt amagtõ*
Roof slope	*Payt amuwi*
Mid wood notches	*Araybu*
Lashings	*Wanaka*

The building is thatched with palms from *Manicaria saccifera*, also harvested during the waning moon phase to prevent attacks by xylophagous insects. The leaves are folded longitudinally and piled starting from the bottom and working toward the top of the roof. The midrib oriented vertically to allow water to run freely. The leaves are lashed to the battens by their midrib. The tighter the leaves, the longer the roof will last. A supplementary layer of palms is placed horizontally along the top to seal the ridge and held in place by wooden sticks stuck into the roof.

### Species used in the composition of building

Inventories of buildings under construction resulted in a total of 960 roundwood pieces, divided between 100 posts (85 permanents and 15 temporaries; see Table 3) and 860 frame pieces (783 permanents and 77 temporaries; see Table 4) in 24 buildings (20 permanent and 4 temporary). Twenty-seven folk wood taxa were recorded (Tables 3 and 4). Among these 24 buildings, 46% (11/24) had a frame made of roundwood only.

**Table 3:** Wood names of the 100 inventoried posts in permanent or temporary buildings (wood ranked according to the total number of citation per use)

Botanic family	Palikur name	Rank	Permanent (total 85)	Temporary (total 15)
Annonaceae	*Pukuu* (*seyne*)	7	2	0
	*Kuukumwi* (*priye*)	7	1	1
	*Kuukumwi* (*seyne*)	8	1	0
Apocynaceae	*Isuu ã*	3	6	0
Chrysobalanceae	*Bukutru gatew*	3	0	6
Erythroxylaceae	*Yawknabui duwõ*	7	2	0
Fabaceae	*Ãjelik*	8	1	0
	*Arey avaini*	8	0	1
	*Miumiu*	8	0	1
	*Wakap*	2	12	0
Goupiaceae	*Pasis*	8	0	1
Lecythidaceae	*Avun*	6	2	0
	*Kwatri (waxriune) seyne*	5	6	0
	*Yawknabwi* (*seyne)*	6	3	0
Myristicaceae	*Wahusi (waxriuno duwõ)*	7	1	0
Myrtaceae	*Awaw*	8	1	0
Olacaceae	*Yawu*	1	43	0
Rubiaceae	*Kinuwup*	7	2	0
Sapotaceae	*Balata kamwi*	8	1	0
	*Kuyau (kamwi*) *duwõ*	4	0	5
	*Tukuyuy kamwi*	8	1	0

**Table 4:** Wood names of the 860 components of frameworks inventoried in permanent or temporary buildings (wood ranked according to the total number of citation per use)

Botanic family	Palikur name	Rank	Permanent (total 783)	Temporary (total 77)	Beam	Tie beam	Ridge piece	Crown post	Common rafter	Batten	Wind brace
Annonaceae	*Kuukumwi* (*priye*)	1	210	11	14	21	13	11	71	84	7
	*Kuukumwi* (*seyne*)	2	230	13	11	17	12	18	68	103	14
	*Pukuu* (*seyne*)	3	200	17	2	3	1	12	27	170	2
	*Kigiksau*	8	10	0	2	3	2	–	3	–	–
Chrysobalanceae	*Inutawviye*	7	10	0	2	–	8	–	–	–	–
	*Bukutru gatew*	9	0	9	–	3	–	2	1	1	2
Ebenaceae	*Miret*	6	0	17	–	–	2	–	10	4	1
Bignoniaceae	*Kwik*	14	1	0	–	–	–	–	–	–	1
Erythroxylaceae	*Yawknabwi duwõ*	10	8	0	–	7	1	–	–	–	–
Goupiaceae	*Pasis*	14	1	0	–	–	–	–	–	1	–
Lecythidaceae	*Avun*	5	11	7	4	7	2	–	1	4	–
	*Kwatri (waxriune) seyne*	10	6	0	–	–	–	–	–	6	–
	*Yawknabwi* (*seyne)*	4	88	0	8	14	7	2	40	16	1
Moraceae	*Pairi (seyne)*	14	0	1	–	–	–	1	–	–	–
Myristicaceae	*Wahusi (waxriuno duwõ)*	12	3	0	–	–	–	–	3	–	–
Myrtaceae	*Awaw*	13	2	0	–	–	1	–	–	–	1
Sapotaceae	*balata (duwõ)*	11	3	0	–	–	–	–	2	1	–
	*Kuyaw (kamwi*) *duwõ*	13	0	2	1	–	–	–	1	–	–

Ethnobotanical walks resulted in 63 folk taxa from 233 herbarium vouchers given by 13 informants, potentially used in buildings (resulting in 36 folk taxa more than in inventories of buildings/of buildings under construction?). To improve the scientific and Palikur taxonomical correlation, we used 261 supplementary herbarium vouchers as well as in-field identification of 175 specimens, giving a total of 699 specimens from 25 botanical families (Table 5).

**Table 5:** Botanical names of the most frequently collected species

Botanic family (main palikur name)	Botanical name^a^	Reference voucher specimen^b^	Correspondence botanical species/ethnospecies	Fidelity level of use^c^	
				Post	Frame
Annonaceae					
*Kigiksaw*	***Guatteria punctata*** (Aubl.) R.A. Howard	CO.80	4/13	0/3	3/3
	*Guatteria wachenheimii* Benoist	MC.392	3/13		
	***Anaxagorea phaeocarpa*** Mart.	CO.281	2/13		
	*Guatteria anteridifera* Scharf & Maas	MC.50	2/13		
*Kuukumwi priye*	***Oxandra asbeckii*** (Pulle) R.E. Fr.	CO.1	16/29	1/6	6/6
	*Guatteria wachenheimii* Benoist	CO.320	4/29		
	*Unonopsis rufescens* (Baill.) R.E. Fr.	CO.65	3/29		
	*Guatteria anteridifera* Scharf & Maas	CO.266	2/29		
	***Pseudoxandra cuspidata*** Maas	MC.663	2/29		
*Kuukumwi seyne*	*Pseudoxandra cuspidata* Maas	CO.13	5/5	0/5	5/5
*Pukuu* (*seyne*)	***Xylopia nitida*** Dunal	CO.40	6/9	0/3	3/3
	*Xylopia cayennensis* Maas	Gr. 1677	3/9		
Apocynaceae					
*Isuu ã*	***Aspidosperma excelsum*** Benth.	MC.24	2/2	1/1	0/1
*Gõngo*	***Geissospermum laeve*** (Vell.) Miers	CO.118	3/3	1/1	1/1
*Pakih etni*	***Parahancornia fasciculata*** (Poir.) Benoist	Gr.3064	4/4	0/2	2/2
Bignoniaceae					
*Kwik*	*Handroanthus serratifolius* (Vahl) S.O.Grose	Gr.3109	2/2		
Burseraceae					
*Ahuwahu*	***Protium opacum*** Swart	L.796	5/19	0/3	3/3
	*Protium tenuifolium* (Engl.) Engl.	Pr.Gr.4336	3/19		
	*Protium apiculatum* Swart	Gr.1583	3/19		
	*Protium morii* D.C. Daly	MC.456	2/19		
*Marinaiwa*	***Protium altsonii*** Sandwith	MC.26	3/13	0/3	3/3
	***Protium gallicum*** D.C. Daly	Gr.2143	3/13		
	*Protium decandrum* (Aubl.) Marchand	MC.109	2/13		
	*Protium trifoliolatum* Engl.	MC.117	2/13		
*Sirasira*	***Protium decandrum*** (Aubl.) Marchand	MC.22	3/14	0/4	4/4
	*Tetragastris panamensis* (Engl.) Kuntze	Gr.1858	2/14		
	*Protium subserratum* (Engl.) Engl.	Gr.3125	2/14		
Chrysobalanaceae					
*Bukutru gatew*	***Licania alba*** (Bernoulli) Cuatrec.	CO.345	12/40	5/5	5/5
	*Couepia caryophylloides* Benoist	CO.356	4/40		
	*Hirtella bicornis* Mart. & Zucc.	CO.293	4/40		
	*Hirtella glandulosa* Spreng.	CO.334	2/40		
	*Licania densiflora* Kleinhoonte	CO.144	2/40		
	*Licania membranacea* Sagot ex Laness.	Pr.Gr.4370	2/40		
*Inutauviye*	***Licania heteromorpha*** Benth.	CO.229	7/16	2/2	2/2
Clusiaceae					
*Kwatri* (*waxriune*) *duwõ*	*Tovomita brevistaminea* Engl.	CO.292	2/10	3/3	3/3
	*Tovomita choisyana* Planch. & Triana	CO.29	2/10		
	*Tovomita* sp. 1	CO.82	2/10		
	*Tovomita* sp. 2	CO.17	2/10		
*Ti*	***Symphonia globulifera*** L.f.	CO.57	4/5	0/2	2/2
*Wakukwa tiranõ*	***Garcinia macrophylla*** Mart.	Gr.2128	3/7	0/1	1/1
	*Garcinia benthamiana* (Planch. & Triana) Pipoly	MC.343	2/7		
	*Garcinia madruno* (Kunth) Hammel	Gr.3119	2/7		
Ebenaceae					
*Miret*	*Diospyros carbonaria* Benoist	MC.70	3/13	0/1	1/1
	*Diospyros dichroa* Sandwith	Gr.3157	3/13		
	*Diospyros cavalcantei* Sothers	MC.46	2/13		
Elaeocarpaceae					
*Waaduk (seyne)*	***Sloanea laxiflora*** Spruce ex Benth.	CO.286	5/5	0/1	1/1
Erythroxylaceae					
*Yawknabwi duwõ*	***Erythroxylum amplum*** Benth.	Gr.Pr.2050	4/4	2/3	3/3
Goupiaceae					
*Pasis*	***Goupia glabra*** Aubl.	CO.18	5/5	2/3	3/3
Ixonanthaceae					
*Yawu wahuyo*	*Cyrillopsis paraensis* Kuhlm.	CO.46	2/2	5/5	0/5
Lauraceae					
*Migukat*	***Licaria martiniana*** (Mez) Kosterm.	MC.650	2/10	0/3	3/3
	***Ocotea percurrens*** Vicent.	Gr.1680	2/10		
*Panawnap*	***Aiouea longipetiolata*** van der Werff	MC.155	2/5	2/3	3/3
*Sedri kamwi*	***Licaria martiniana*** (Mez) Kosterm.	CO.231	3/7	1/4	4/4
*Wen*	***Ocotea guianensis*** Aubl.	Gr.2068	7/8	1/2	2/2
	Lauraceae sp. 3	CO.170	1/8		
Lecythidaceae					
*Avun*	***Eschweilera coriacea*** (DC.) S.A. Mori	Gr.Pr.1990	5/38	5/6	6/6
	***Lecythis persistens*** Sagot	CO.27	5/38		
	*Eschweilera grandiflora* (Aubl.) Sandwith	MC.212	4/38		
	*Eschweilera sagotiana* Miers	CO.254	4/38		
	*Eschweilera apiculata* (Miers) A.C.Sm.	MC.116	3/38		
	*Lecythis holcogyne* (Sandwith) S.A. Mori	CO.148	3/38		
	*Eschweilera chartaceifolia* S.A. Mori	MC.321	2/38		
*Kwatri waxriune* (*seyne*)	***Lecythis poiteaui*** O. Berg	CO.19	6/21	3/3	3/3
	*Eschweilera* cf. *simiorum* (Benoist) Eyma	CO.160	3/21		
	*Eschweilera chartaceifolia* S.A. Mori	MC.60	2/21		
Leguminosae					
*Ãjelik*	***Dicorynia guianensis*** Amshoff	Gr.3057	10/10	3/3	3/3
*Kaybune ã*	***Zygia racemosa*** (Ducke) Barneby & J.W. Grimes	CO.4	3/3	1/1	1/1
*Miumiu*	***Inga paraensis*** Ducke	Gr.3175	3/13	1/3	3/3
	*Inga capitata* Desv.	Gr.JLG.3216	2/13		
	*Inga* sp. 15	CO.332	2/13		
*Sakeg* (*kamwi*)	*Hymenolobium flavum* Kleinhoonte	Gr.3067	2/13	0/2	2/2
	*Parkia nitida* Miq.	Gr.3030	2/13		
*Ã danõ*	***Bocoa prouacensis*** Aubl.	Gr.3269	5/5	0/2	2/2
*Wakap*	***Vouacapoua americana*** Aubl.	Gr.3011	16/16	4/4	2/4
*Wap*	***Eperua falcata*** Aubl.	CO.52	4/12	4/4	4/4
	*Dialium guianense* (Aubl.) Sandwith	Gr.1766	2/12		
	*Eperua grandiflora* (Aubl.) Benth.	Gr.3082	2/12		
	*Macrolobium bifolium* (Aubl.) Pers.	Gr.1662	2/12		
*Yuhumwi*	***Pentaclethra macroloba*** (Willd.) Kuntze	Gr.1643	3/5	1/1	1/1
*Wakukwa adava*	***Gustavia augusta*** L.	MFP.1365	4/8	1/1	1/1
	*Lecythis zabucajo* Aubl.	Gr.3078	2/8		
Melastomataceae					
*Ahayumna*	***Mouriri nervosa*** Pilg.	Gr.3153	6/8	3/5	5/5
	*Mouriri francavillana* Cogn.	L.836	2/8		
*Avitkat*	***Mouriri francavillana*** Cogn.	DD.14	3/5	1/3	3/3
	*Mouriri crassifolia* Sagot	CO.323	2/5		
*Timuvukti*	***Mouriri sagotiana*** Triana	Gr.1627	7/8	0/3	3/3
Moraceae					
*Impitit waxriune*	***Maquira guianensis*** Aubl.	CO.311	3/4	0/1	1/1
*Pairi*	***Brosimum rubescens*** Taub.	CO.172	6/16	1/2	2/2
	*Brosimum guianense* (Aubl.) Huber ex Ducke	MC.6	3/16		
	*Trymatococcus oligandrus* (Benoist) Lanj.	Gr.3143	3/16		
*Pakaad*	***Bagassa guianensis*** Aubl.	Gr.1655	2/2	1/1	1/1
*Tukwangu*	*Helicostylis tomentosa* (Poepp. & Endl.) J.F.Macbr.	MC.508	2/7	0/2	2/2
	*Naucleopsis guianensis* (Mildbr.) C.C. Berg	Gr.3148	2/7		
	*Pseudolmedia laevis* (Ruiz & Pav.) J.F.Macbr.	CO.227	2/7		
Myristicaceae					
*Wahusi* (*waxriune*)	***Virola michelii*** Heckel	Gr.3036	7/20	0/3	3/3
	*Iryanthera hostmannii* (Benth.) Warb.	Gr.1754	3/20		
	*Iryanthera sagotiana* (Benth.) Warb.	Pr.Gr.4392	3/20		
	*Virola kwatae* Sabatier	MC.20	2/20		
	*Virola multicostata* Ducke	MC.417	2/20		
	*Virola surinamensis* (Rol. ex Rottb.) Warb.	DD.15	2/20		
Myrtaceae					
*Awaw*	***Myrciaria floribunda*** (H. West ex Willd.) O. Berg	Gr.3002	4/9	2/3	3/3
*Inam etni*	***Eugenia coffeifolia*** DC.	CO.287	6/22	4/5	5/5
	*Eugenia patrisii* Vahl	Gr.3091	3/22		
	*Myrcia decorticans* DC.	MC.49	3/22		
	*Eugenia* sp.FG13 (Holst)	MC.77	2/22		
*Kagegut*	***Myrcia fallax*** (Rich.) DC.	Gr.1802	2/3	1/1	1/1
Ochnaceae					
*Kwatri waxriune* (*duwõ*)	*Lacunaria* cf. *jenmanii* (Oliv.) Ducke	CO.353	2/6	1/1	1/1
	*Quiina sessilis* Choisy	MC.503	2/6		
Olacaceae					
*Aneku*	***Ptychopetalum olacoides*** Benth.	CM.1060	5/5	0/1	1/1
*Yawu*	***Minquartia guianensis*** Aubl.	Gr.3007	5/5	5/5	0/5
Rubiaceae					
*Ã wakaha*	*Ferdinandusa paraensis* Ducke	MC.480	1/2	0/1	1/1
	*Ferdinandusa* sp. 1	CO.299	1/2		
*Kinuwup*	***Duroia eriopila*** L.f.	Gr.1760	2/4	1/2	1/2
Sapindaceae					
*Mbagwi*	***Cupania scrobiculata*** Rich.	CO.147	4/9	0/2	2/2
	*Matayba* sp. undet	MC.235	2/9		
*Tuu*	*Talisia carinata* Radlk.	Gr.1585	1/5	0/3	3/3
	*Talisia megaphylla* Sagot	Gr.1753	1/5		
	*Talisia mollis* Kunth ex Cambess.	CO.185	1/5		
	*Talisia* sp. undet	MC.588	1/5		
	*Toulicia elliptica* Radlk.	L.788	1/5		
Sapotaceae					
*Balata* (*duwõ*)	***Manilkara huberi*** (Ducke) A. Chev.	Gr.3035	3/4	2/2	2/2
*Kuyaw kamwi*	***Pouteria decorticans*** T.D. Penn.	MC.83	23/36	6/6	6/6
	*Pouteria gongrijpii* Eyma	Pr.Gr.4277	7/36		
	*Pouteria filipes* Eyma	Gr.3177	2/36		
	*Pouteria singularis* T.D.Penn.	MC.160	2/36		
*Tukuyuy kamwi*	***Pouteria jariensis*** Pires & T.D.Penn.	Gr.3183	2/4	2/2	2/2
*Uu kamwi*	***Micropholis cayennensis*** T.D.Penn.	Gr.3059	5/56	8/8	8/8
	***Pouteria gongrijpii*** Eyma	Pr.Gr.4293	5/56		
	***Pouteria torta*** (Mart.) Radlk.	CO.106	5/56		
	*Pouteria rodriguesiana* Pires & T.D.Penn.	Gr.3130	4/56		
	*Pouteria guianensis* Aubl.	CO.252	4/56		
	*Pouteria aubrevillei* Bernardi	MC.108	3/56		
	*Pouteria decorticans* T.D. Penn.	CO.33	3/56		
	*Pouteria macrocarpa* (Mart.) D.Dietr.	CO.111	3/56		
	*Pouteria singularis* T.D.Penn.	CO.290	3/56		
	*Micropholis guyanensis* (A.DC.) Pierre	Gr.1749	2/56		
	*Pouteria hispida* Eyma	CO.154	2/56		
	*Pouteria reticulata* (Engl.) Eyma	MC.699	2/56		
Siparunaceae					
*Avakni avak*	***Siparuna pachyantha*** A.C. Sm.	Gr.3172	2/2	1/1	1/1
*Yahiwemna*	***Siparuna cristata*** (Poepp. & Endl.) A.DC.	CO.284	3/6	0/1	1/1
	*Siparuna guianensis* Aubl.	Gr.1850	2/6		

^a^In bold, the most representative species

^b^Deposited at the CAY herbarium, French Guiana

^c^Ratio calculated as the no. of given botanic species vouchers/total vouchers for this ethnospecies (total vouchers 669). Fidelity level of use is calculated according to [23]

In Palikur ethnobotany, correlations between botanical and folk taxa are particularly difficult to establish. Only 25% of the folk species matched a botanical species in a one-to-one correspondence, following Berlin’s nomenclature (1993). Three quarters of the folk species refer to groups of botanical species, illustrating a pattern Berlin [9] describes as under differentiation: 25% of the total refer to different species in a single genus, 48% refer to species in different genera in the same botanical family, and 2% refer to species in different families (Table 5).

#### Wood preferences and selection criteria

##### Posts

Field surveys revealed that 65% (55/85) of the woods cited as being used for posts in permanent buildings belong to two botanical species (Table 3): *Minquartia guianensis*, 51% (43/85), and *Vouacapoua americana*, 14% (12/85). Young, straight, and fluted trunks of *M. guianensis* and *V. americana* are highly appreciated by the Palikur for their hardness, their durability, and their resistance to rot and to wood-eating insects. The wood from *V. americana* is so highly valued by them that it has become the prototype species for posts: *wakap* refers both to the term “post” and to the species. Wood from the genera *Eschweilera* and *Lecythis* in the Lecythidaceae family account for 13% (11/85) of the permanent posts, even though the Palikur are aware they are less durable as far as rotting and xylophages are concerned. *Aspidospermum excelsum* wood is cited for 7% (6/85) of the permanent posts and is also well known for crafting paddles. The hard heavy wood from several Chrysobalanaceae species (*Licania*, *Hirtella*, *Couepia*, named *bukutru gatew*) is also used but to a lesser extent, due to their tendency to split when dropped and because their bark is difficult to peel off. Several *Pouteria* (*kuyaw kamwi duwõ* and *tukuyuy kamwi*) are used for posts as second choice (7%; 6/85 for both).

##### Beams and rafters

Most of the elements of the permanent building frame come from two families: Annonaceae (79%; 681/860) and Lecythidaceae (13%; 112/860). Among the Annonaceae, the most frequently used are *Oxandra asbeckii*, *Pseudoxandra cuspidata*, *Xylopia nitida*, and *X. cayennensis*.

The two *Xylopia* species are preferentially used for thin pieces of the framework, and *pukuu* accounts for 78% of the battens. Among the Lecythidaceae, *Lecythis persistens*, *Eschweilera* cf. *coriacea*, *E. sagotiana*, and *L. poiteaui* are the most frequently used. The Palikur justify the use of Annonaceae because of their long straight trunks that are ideal for long span beams. Unlike the wood from Lecythidaceae, the Annonaceae wood does not split, either when being felled or while drying. It is also lighter and more flexible than other woods of the same size, making framing easier and enabling the construction of apses. After drying, *kuukumwi* wood hardens and becomes very resistant. Annonaceae wood is generally perceived as being more resistant to xylophagous insects than Lecythidaceae wood whose sapwood is seriously attacked by wood-eating beetle larvae.

Nevertheless, the two families share the advantage of having fibrous bark, which is easily peeled off in wide longitudinal strips, thereby facilitating preparation. Easy debarking is a technical and esthetic selection criterion for building use. Indeed, 90% (860/960) of the pieces of wood counted were bare. Debarking is almost systematic for permanent buildings (98%; 850/868), while less attention is paid to debarking in the case of temporary buildings (11%; 10/92). The wood for provisional buildings is selected with less care. Availability in the vicinity of the building site is more important than the technical qualities of the wood, meaning brittle wood or wood that is hard to debark is also used. This is the case for *Diospyros* spp., which accounted for 22% (17/77) of all the pieces of wood used in temporary buildings counted.

#### Lash and thatch materials

Today, nails are increasingly used to assemble frame elements. Only two out of 25 buildings were made from roundwood lashed together without nails. Lashings are made of aerial roots of two hemi-epiphytic species: *Thoracocarpus bissectus* (Cyclanthaceae) and *Heteropsis flexuosa* (Araceae). Roots of *T. bissectus* are considered to be stronger than those of *H. flexuosa*, but they have to be soaked underwater for 2 to 3 weeks before being peeled and used, while the latter are easier to peel [15]. The roots are split longitudinally before use.

Among the 25 buildings inventoried, 23 have a thatched roof and two have corrugated iron roofs. The thatching material is always made from the palms of *M. saccifera* (Table 6). The palms are mainly lashed to the battens (52%; 12/23). Lashings are mainly made from *T. bissectus* (Table 6), followed by *H. flexuosa* or from splints of *Ischnosiphon obliquus* (Marantaceae). Today, these techniques are slowly being replaced by nails (48%; 11/23).

**Table 6:** Thatch and latch materials

Botanic family	Palikur name	Botanic determination (herbarium voucher no.)
Cyclanthaceae	*Akuywa*	*Thoracocarpus bissectus* (Vell.) Harling (DD.11)
Marantaceae	*Wevri*	*Ischnosiphon obliquus* (Rudge) Körn. (DD.16)
Araceae	*Tiravui*	*Heteropsis flexuosa* (Kunth) G.S.Bunting (Gr.JLG.3231)
Arecaceae	*Tuuvan*	*Manicaria saccifera* Gaertn.^a^
	*Isaw*	*Mauritia flexuosa* L.f.^a^
	*Issuvan*	*Geonoma baculifera* (Poit.) Kunth^a^

^a^Species not collected for herbarium voucher in this project

It is noteworthy that the use of the small *Geonoma baculifera*, which is widespread in French Guiana, is well known to the Palikur, but their palms only measure 60 × 20 cm, whereas *Manicaria* palms measure more than 300 × 100 cm, so *Geonoma baculifera* is only very occasionally used in our study area (Additional file 1).

### Perception of traditional housing

Nearly half the people who expressed their opinion on this point (48%; 10/21) consider the roundwood housing to be more comfortable from a bioclimatic point of view than concrete buildings. Roundwood housing optimizes ventilation of the structure and allows smoke to escape, which is why the Palikur use it for daily tasks in addition to their living house. It is nevertheless described as cold to sleep in at night. Others use roundwood to build because it is free, as the raw material is collected in neighboring forests without the need for technical equipment (38%; 8/21), for its appearance (33%; 7/21), and lastly for its convenience (19%; 4/21). It should be noted that 84% (33/39) of the Palikur interviewed have no other income than minimum social benefits. But they do have regular access to forest products as the vast majority still practice slash and burn agriculture, fishing, hunting, or harvesting in the forest (94%; 37/39).

## Discussion

### Building techniques

The building techniques and materials used by the Palikur are common to many Amazonian peoples [24, 25, 43], but they are changing with the liberal economic system and access to consumer goods [39]. Settlement influences housing structure in two main ways. The first is the search for buildings that last longer than the former houses, which have a 15-year lifespan. Nails and corrugated iron roofs are thus increasingly replacing lashing and *M. saccifera* palm thatch. The second is pressure on the natural resources as emblematic species, for example, *V. americana* posts are said to be becoming rarer. The logistic effort and the need for a pick-up to harvest building material is greater today. Timber wood, which is more accessible, has become an attractive option. More than just the esthetic dimension, these changes imply a real loss of ecological knowledge and technological know-how. As it has been observed for basketry [15], social changes and schooling may explain the decrease in knowledge transmission.

### Wood selection

Despite the many changes to roundwood housing, the Palikur still have vast knowledge of the materials used in traditional building. Our quantitative results reveal clear preferences for particular species depending on the planned use, with selection based on technical criteria, above all durability. Durability is the most important requirement for woods used for posts, due to their higher exposure. A comparison of our results with reports in the literature underlines the similarities in the characteristics of construction wood among many Amazonian Amerindians [5, 11, 17, 42–44, 52, 56].

#### Posts

Like the Palikur, the Brazilian Waimiri-Atroari make posts from *Minquartia guianensis* (Olacaceae) [44, 62] due to its very high resistance to rot and to xylophagous insects. Its use is also mentioned among the Shuar [8] and the Cayapa in Ecuador [6] and by the Boni Maroons in French Guiana [21]. This species is so highly valued for posts among the Brazilian Tembé and Ka’apor that burning it is prohibited, with a risk of death in the village [55]. This wood is so durable that remains of *M. guianensis* posts were found in a more than 3000-year-old pre-Columbian house discovered at an archeological site [59]. Woods used for posts by the Yanomami are not the same as the wood preferred by the Palikur, but the two use the same criteria, durability, and resistance. The main species are *Manilkara huberi* and various *Pouteria* spp*.* (Sapotaceae), also used as a second choice by the Waimiri-Atroari and are occasionally used by the Palikur, as well as *Centrolobium paraense* (Leguminosae) [42]. The use of the long-lasting wood of *V. americana* and of *Pouteria decorticans* for posts is also mentioned among Boni Maroons [21]. The wood of some species belonging to the families Chrysobalanceae and Lecythidaceae is found among the Bolivian Tsimane’ (*Hirtell*a spp. and *Licania* spp.) [56], the Kalin’a in Guyana (*Couepia* spp., *Eschweilera* spp. and *Lecythis* spp.) [65], or as a substitute for *M. guianensis* among the Waimiri-Atroari (several *Licania* and *Eschweilera* including *E. coriacea*). Yanomami also use the genus *Couepia* for secondary posts [43] also occasionally used by the Palikur. Surprisingly, the Palikur appear to not use Myrtaceae much. The wood of these trees is extremely hard and is favored for the thin external posts in roundhouses in the Guiana shield highlands where the Yanomami live and where the Myrtaceae family is very common [43]. The fact that this family, which is used by the Teko in French Guiana (Odonne and Davy, unpublished data) and by the Kali’na (Grenand, unpublished data), was rarely cited by the Palikur could be due to their low density in the study area.

#### Beams and rafters

Like the Palikur, the Yanomami and Waimiri-Atroari also preferentially use the Annonaceae wood (*Xylopia* spp., *Guatteria* spp., *Duguetia* spp.) for frames [42–44]. The trees belonging to this family are considered to make straight, long range beams. *Xylopia* species are actually described as following the Roux architectural model, with an orthotropic, slender trunk, few ramifications, and plagiotropic branches [37, 49], and are therefore a very good building wood. The wood of the genera *Eschweilera* (Lecythidaceae) and *Pouteria* (Sapotaceae) is also used for beams by the Waimiri-Atroari [44]. Urubu Ka’apor use a wide array of species for the framework, i.e., *Pouteria* spp., *Duguetia* spp., *Guatteria* spp., *Licania* spp., and *Eschweilera* spp., but prefer the durable wood of *Licania* (Chrysobalanceae) or *Eschweilera* species due to their resistance to xylophagous insects [5]. In Bolivia, the Tacana also select Annonaceae (*Guatteria* spp., *Unonopsis* spp., *Xylopia* spp.) and Chrysobalanceae (*Hirtell*a spp. and *Licania* spp.) for beams and rafters [17], as do the Caboclo from the Capim River in Brazil [62]. In Guyana, the wood of several Annonaceae (*Xylopia* spp., *Anaxagorea* spp., *Duguetia* spp., *Guatteria* spp., and *Unonopsis* spp.) is also appreciated for the framework, as are those of *Eschweilera* or *Lecythis* species, but to a lesser extent [65].

### Convergence of evidence-based technological knowledge

These similarities suggest a convergence that could be due to some technological properties of the species. This is illustrated by the high resistance against rot and xylophagous insects of the species used for the posts, for which the quality of the material is more important. The use of *Xylopia* and *Pseudoxandra* genera for beams or rafters throughout Amazonia is explained by their straight shape, resistance (poorly documented), and easy debarking. This last criterion might seem above all esthetic, but the Palikur explicitly mentioned that debarked wood is more resistant to xylophagous insects. Whether related to technological factors or not, this propensity for easily peeled woods throughout Amazonia is a very distinctive illustration of cultural convergence. Laboratory tests are desirable to understand these cultural selection processes [57].

The similarities are even more striking in lashing materials. Aerial roots of *Heteropsis* spp. are the most widely used lashing material in Amazonian buildings [5, 8, 19, 21, 29, 42, 44, 65] and are more extensively used than the second choice *T. bissectus* [42, 48, 56, 65]. There is less consensus for roof thatching, as aside from the Palikur, only the Arawak in Guyana also use *M. saccifera* palms [65], very likely due to the limited geographical distribution of this species, which is mainly restricted to the swamps and low drainage areas of the Guiana shield [26]. The use of *Geonoma* palms as thatching material (here *G. baculifera*) is much more widespread in Amazonia [5, 21, 22, 29, 42, 48, 56, 65, 66].

### Resource availability

The availability of a resource inevitably influences its use. For example, many Amazonian groups decide on the location of a village based on the availability of “stationary” resources, such as thatching material. Testing whether the use of some species depends on their availability for building would have required extensive botanical inventories around Regina and Saint-Georges de l’Oyapock, so we will limit our discussion here to conjecture. Nevertheless, the influence of the availability of a species appears to depend to a great extent on the type of building planned: a light building with a projected life span of 2 or 3 years, in a slash and burn field, for example, will be preferentially built using species that grow in the immediate vicinity despite their inferior quality. On the other hand, a permanent collective building, with more social value, will be the object of a more rigorous material selection. The fine analysis of the balance between quality and availability would need more detailed research.

Hyper-dominance of trees belonging to the Lecythidaceae family and their pan-Amazonian distribution (including *Eschweilera coriacea*) is certainly one of the factors that explain their frequent use for building by the Palikur and other Amazonian peoples [64]. The Annonaceae family, held in high esteem for the framework, is also abundant throughout Amazonia. Moreover, several species of *Guatteria* and *Xylopia* (also used for the framework by the Wayãpi in French Guiana) are fostered by anthropization and are thus found close to the villages located in secondary forests [30, 65].

### Why does roundwood building persist?

Although housing has undergone significant changes, many communities are willing to conserve traditional buildings or to start using them again for ceremonial functions, as a way to claim their cultural identity, by the practice of rituals, dances, and craftwork, and as a place of social networking and community building, even if some authors consider that these activities previously took place outdoors [46]. Today, activities such as basketry, music, or dances are important ways to affirm their identity among French Guiana Amerindians [15].

Simultaneously, the Palikur have become aware of the mismatch between modern housing and their traditional way of life [10]. Roundwood buildings provide natural ventilation, lowering the high tropical temperatures and the ambient moisture. Their low cost is also an advantage for people who only have a limited income. The esthetic dimension of these buildings should not be ignored, nor is the well-being they insure through the creation of social ties. The people we interviewed did not think modern housing is well suited to the still largely collective way of life of the Palikur.

The loss of knowledge observed in some cases is offset by regular exchanges between the Palikur in French Guiana and in Brazil, the latter often being perceived as more skillful and knowledgeable about cultural and natural aspects. In French Guiana, the Palikur still maintain strong interactions with the forest, as 71% of people interviewed by Sevelin-Radiguet [60] said they go hunting regularly, 65% harvest woody forest products, and 88% harvest non-woody products. Although unemployment may encourage these practices as alternative sources of income, they more likely reflect the persistence of the former way of life that was closer to nature.

## Conclusions

From an ethnobotanical point of view, the Palikur use only a small fraction of the ca. 1600 ligneous species present in French Guiana [45]. This is evidence for the existence of rigorous selection criteria, technological as much as cultural. For posts, hard woods are preferred, as they are known to be resistant to rot and to xylophage activity, which are key requirements for durability, since the posts are in direct contact with the ground and the framework weighs hundreds of kilograms. The *yawu*, *Minquartia guianensis*, and the *wakap*, *Vouacapoua americana*, are their emblematic species, followed by many Lecythidaceae, Leguminosae, and Sapotaceae also observed on both sides of the Amazon for this use, testifying to a consensus in technical selection criteria among Amazonian Amerindians. The main species used for the framework belong to the Annonaceae family, genera *Xylopia*, *Guatteria*, or *Duguetia*. Lecythidaceae, and notably the *Eschweilera* genus, are also highly esteemed as elsewhere in Amazonia. The technical properties of these woods, and notably their use as roundwood, remain to be analyzed. It is nevertheless highly probable that a long-term selection process led to the identification of species that combine useful mechanical properties, high resistance against rot and xylophage activity, and ease of use during construction. The ease of stripping the bark from the trunk is one of the most important criteria. Combining the useful with the pleasant, this technical and esthetic choice echoes the selection of fluted trunks, which are said to be nicer, for posts.

The choice between abundance in the vicinity and technical criteria appears to depend on the planned use of the building. Woods are selected much more carefully for long-lasting buildings, although they often require more complex logistics. Today, this is offset by the increasing use of squared timber, as species that were difficult or impossible to use as roundwood are now more easily available. Understanding these trade-offs more precisely would require a more precise survey focused on the one hand on the abundance and ecological distribution of available species (both those used as roundwood and those used as square timber) and on the other hand on the criteria of choice that influence construction processes.

From a socio-cultural point of view, the persistence of roundwood houses is first explained by the attachment of the Palikur to the comfort they provide in a tropical climate. Their low construction cost and esthetic considerations are nevertheless equally cited. Continuing to build roundwood houses and going into the forest to harvest useful materials for their construction may also be a way for the Palikur to claim their cultural identity by asserting their indigenousness and the superiority of a particular way of life. Like other cultural activities, such as basketry, highly esteemed by the Palikur [15], maintaining these century-old practices is a form of cultural resistance against a modern world prone to imposing its technical standards.

## Additional file

Additional file 1: Full list of ethnospecies and their botanic correspondences. (DOCX 217 kb)
