# Supplementary material for: Palikur traditional roundwood construction in eastern French Guiana: ethnobotanical and cultural perspectives
Source: J Ethnobiol Ethnomed. 2018 Apr 24;14:28. doi: 10.1186/s13002-018-0226-7 (PMC5916587; doi:10.1186/s13002-018-0226-7)
Supplement: Supplementary file 1 — Full list of ethnospecies and their botanic correspondences. (DOCX 217 kb) [file 13002_2018_226_MOESM1_ESM.docx]

Additional file 1: Full list of ethnospecies and their botanic correspondences.

***Legend***. ^a^ In bold, the most representative species. ^b^ Deposited at the CAY herbarium, French Guiana. ^c^ Ratio calculated as the N° of a botanic species vouchers/total vouchers for this ethnospecies (total vouchers: 669) Fidelity level of use calculated according to Friedman et *al.*, 1986.

| **Botanic family**  Main palikur name | Botanical name ^a^ | Reference voucher specimen^b^ | Correspondence botanical species/  ethnospecies^c^ | **Fidelity level of use** | |
| --- | --- | --- | --- | --- | --- |
|  |  |  |  | **Post** | **Frame** |
| **Annonaceae** |  |  |  |  |  |
| *Kigiksaw* | ***Guatteria punctata*** (Aubl.) R.A. Howard | CO.80 | 4/13 | **0/3** | **3/3** |
|  | *Guatteria wachenheimii* Benoist | MC.392 | 3/13 |  |  |
|  | ***Anaxagorea phaeocarpa*** Mart. | CO.281 | 2/13 |  |  |
|  | *Guatteria anteridifera* Scharf & Maas | MC.50 | 2/13 |  |  |
|  | *Guatteria guianensis* (Aubl.) R.E.Fr. | CO.22 | 1/13 |  |  |
|  | *Guatteria ouregou* (Aubl.) Dunal | Gr.2079 | 1/13 |  |  |
| *Kuukumwi* (*priye*) | ***Oxandra asbeckii*** (Pulle) R.E. Fr. | CO.1 | 16/29 | **1/6** | **6/6** |
|  | *Guatteria wachenheimii* Benoist | CO.320 | 4/29 |  |  |
|  | *Unonopsis rufescens* (Baill.) R.E. Fr. | CO.65 | 3/29 |  |  |
|  | *Guatteria anteridifera* Scharf & Maas | CO.266 | 2/29 |  |  |
|  | *Pseudoxandra cuspidata* Maas | MC.663 | 2/29 |  |  |
|  | *Fusaea longifolia* (Aubl.) Saff. | CO.146 | 1/29 |  |  |
|  | *Unonopsis stipitata* Diels | Gr.3161 | 1/29 |  |  |
| *Kuukumwi* (*seine*) | ***Pseudoxandra cuspidata*** Maas | CO.13 | 5/5 | **0/5** | **5/5** |
| *Pukuu* (*seine*) | ***Xylopia nitida*** Dunal | CO.40 | 6/9 | **0/3** | **3/3** |
|  | *Xylopia cayennensis* Maas | Gr.1677 | 3/9 |  |  |
| **Apocynaceae** |  |  |  |  |  |
| *Isuu ã* | ***Aspidosperma excelsum***Benth. | MC.24 | 2/2 | **1/1** | **0/1** |
| *Gõngo* | ***Geissospermum laeve*** (Vell.) Miers | CO.118 | 3/3 | **1/1** | **1/1** |
| *Pakih etni* | ***Parahancornia fasciculata*** (Poir.) Benoist | Gr.3064 | 4/4 | **0/2** | **2/2** |
| **Bignoniaceae** |  |  |  |  |  |
| *Kwik* | *Handroanthus serratifolius*(Vahl) S.O.Grose | Gr.3109 | 2/2 |  |  |
| **Burseraceae** |  |  |  |  |  |
| *Ahuwahu* | ***Protium opacum*** Swart | L.796 | 5/19 | **0/3** | **3/3** |
|  | *Protium tenuifolium* (Engl.) Engl. | Pr.Gr.4336 | 3/19 |  |  |
|  | *Protium apiculatum* Swart | Gr.1583 | 3/19 |  |  |
|  | *Protium morii* D.C. Daly | MC.456 | 2/19 |  |  |
|  | *Protium cuneatum* Swart | Gr.3050 | 1/19 |  |  |
|  | *Protium decandrum* (Aubl.) Marchand | CO.74 | 1/19 |  |  |
|  | *Protium sagotianum* Marchand | Pr.Gr.4315 | 1/19 |  |  |
|  | *Tetragastris hostmannii*(Engl.) Kuntze | CO.5 | 1/19 |  |  |
|  | *Tetragastris panamensis* (Engl.) Kuntze | MC.169 | 1/19 |  |  |
|  | *Trattinnickia demerarae* Sandwith | Gr.Pr.2023 | 1/19 |  |  |
| *Marinaiwa* | ***Protium altsonii*** Sandwith | MC.26 | 3/13 | **0/3** | **3/3** |
|  | ***Protium gallicum*** D.C. Daly | Gr.2143 | 3/13 |  |  |
|  | *Protium decandrum* (Aubl.) Marchand | MC.109 | 2/13 |  |  |
|  | *Protium trifoliolatum* Engl. | MC.117 | 2/13 |  |  |
|  | *Protium* sp. 5 | CO.73 | 1/13 |  |  |
|  | *Protium* sp. 6 | CO.151 | 1/13 |  |  |
|  | *Protium* sp. undet. | MC.304 | 1/13 |  |  |
| *Sirasira* | ***Protium decandrum*** (Aubl.) Marchand | MC.22 | 3/14 | **0/4** | **4/4** |
|  | *Tetragastris panamensis* (Engl.) Kuntze | Gr.1858 | 2/14 |  |  |
|  | *Protium subserratum* (Engl.) Engl. | Gr.3125 | 2/14 |  |  |
|  | *Protium gallicum*D.C. Daly | CO.123 | 1/14 |  |  |
|  | *Protium spruceanum*(Benth.) Engl. | MC.146 | 1/14 |  |  |
|  | *Protium* sp. 1 | CO.6 | 1/14 |  |  |
|  | *Protium* spp. undet. | MC.106 | 4/14 |  |  |
| **Chrysobalanaceae** |  |  |  |  |  |
| *Bukutru gatew* | ***Licania alba*** (Bernoulli) Cuatrec. | CO.345 | 12/40 | **5/5** | **5/5** |
|  | *Couepia caryophylloides* Benoist | CO.356 | 4/40 |  |  |
|  | *Hirtella bicornis* Mart. & Zucc. | CO.293 | 4/40 |  |  |
|  | *Hirtella glandulosa* Spreng. | CO.334 | 2/40 |  |  |
|  | *Licania densiflora* Kleinhoonte | CO.144 | 2/40 |  |  |
|  | *Licania membranacea* Sagot ex Laness. | Pr.Gr.4370 | 2/40 |  |  |
|  | *Couepia guianensis* Aubl. | CO.246 | 1/40 |  |  |
|  | *Couepia joaquinae* Prance | CO.347 | 1/40 |  |  |
|  | *Couepia parillo* DC. | CO.96 | 1/40 |  |  |
|  | *Hirtella hispidula* Miq. | CO.328 | 1/40 |  |  |
|  | *Licania canescens* Benoist | MC.201 | 1/40 |  |  |
|  | *Licania* cf. *micrantha* Miq. | CO.36 | 1/40 |  |  |
|  | *Licania cyathodes* Benoist | Gr.1800 | 1/40 |  |  |
|  | *Licania hypoleuca* Benth. | Gr.JLG.3207 | 1/40 |  |  |
|  | *Licania kunthiana* Hook.f. | Gr.3069 | 1/40 |  |  |
|  | *Licania laxiflora* Fritsch | Gr.3009 | 1/40 |  |  |
|  | *Licania ovalifolia* Kleinh. | CO.297 | 1/40 |  |  |
|  | *Licania robusta* Sagot | CO.265 | 1/40 |  |  |
|  | *Licania* sp. undet. | MC.310 | 1/40 |  |  |
|  | *Licania* sp. 6 Mol et Sab | CO.109 | 1/40 |  |  |
| *Inutawviye* | ***Licania heteromorpha*** Benth. | CO.229 | 7/16 | **2/2** | **2/2** |
|  | *Licania amapaensis* Prance | MC.89 | 1/16 |  |  |
|  | *Couepia guianensis* Aubl. | MC.199 | 1/16 |  |  |
|  | *Hirtella bicornis* Mart. & Zucc. | CO.329 | 1/16 |  |  |
|  | *Hirtella hispidula* Miq. | CO.329-BIS | 1/16 |  |  |
|  | *Hirtella suffulta* Prance | MC.320 | 1/16 |  |  |
|  | *Licania cyathodes* Benoist | CO.165 | 1/16 |  |  |
|  | *Licania divaricata* Benth. | MC.639 | 1/16 |  |  |
|  | *Licania laevigata* Prance | MC.554 | 1/16 |  |  |
|  | *Licania latifolia* Benth. ex Hook.f. | Gr.3038 | 1/16 |  |  |
| **Clusiaceae** |  |  |  |  |  |
| *Kwatri* (*waxriune*) *duwõ* | *Tovomita brevistaminea*Engl. | CO.292 | 2/10 | **3/3** | **3/3** |
|  | *Tovomita choisyana*Planch. & Triana | CO.29 | 2/10 |  |  |
|  | *Tovomita* sp. 2 | CO.17 | 2/10 |  |  |
|  | *Tovomita* sp. 1 | CO.82 | 2/10 |  |  |
|  | *Tovomita* sp. undet. | Gr.3132 | 1/10 |  |  |
|  | *Tovomita* sp. undet. | Gr.3184 | 1/10 |  |  |
| *Ti* | ***Symphonia globulifera*** L.f. | CO.57 | 4/5 | **0/2** | **2/2** |
|  | *Symphonia* sp.1 | Gr.3041 | 1/5 |  |  |
| *Wakukwa tiranõ* | ***Garcinia macrophylla*** Mart. | Gr.2128 | 3/7 | **0/1** | **1/1** |
|  | *Garcinia benthamiana* (Planch. & Triana) Pipoly | MC.343 | 2/7 |  |  |
|  | *Garcinia madruno* (Kunth) Hammel | Gr.3119 | 2/7 |  |  |
| **Ebenaceae** |  |  |  |  |  |
| *Miret* | *Diospyros carbonaria* Benoist | MC.70 | 3/13 | **0/1** | **1/1** |
|  | *Diospyros dichroa* Sandwith | Gr.3157 | 3/13 |  |  |
|  | *Diospyros cavalcantei* Sothers | MC.46 | 2/13 |  |  |
|  | *Diospyros capreifolia*Mart. ex Hiern | MC.359 | 1/13 |  |  |
|  | *Diospyros guianensis*(Aubl.) Gürke | Pr.Gr.4267 | 1/13 |  |  |
|  | *Diospyros* spp. undet | MC.181 | 3/13 |  |  |
| **Elaeocarpaceae** |  |  |  |  |  |
| *Waaduk (seyne)* | ***Sloanea laxiflora*** Spruce ex Benth. | CO.286 | 5/5 | **0/1** | **1/1** |
| **Erythroxylaceae** |  |  |  |  |  |
| *Yawknabwi* (*duwõ)* | ***Erythroxylum amplum*** Benth. | Gr.Pr.2050 | 4/4 | **2/3** | **3/3** |
| **Goupiaceae** |  |  |  |  |  |
| *Pasis* | ***Goupia glabra*** Aubl. | CO.18 | 5/5 | **2/3** | **3/3** |
| **Ixonanthaceae** |  |  |  |  |  |
| *Yawu wahuyo* | *Cyrillopsis* *paraensis* Kuhlm. | CO.46 | 2/2 | **5/5** | **0/5** |
| **Lauraceae** |  |  |  |  |  |
| *Migukat* | ***Licaria martiniana*** (Mez) Kosterm. | MC.650 | 2/10 | **0/3** | **3/3** |
|  | ***Ocotea percurrens*** Vicent. | Gr.1680 | 2/10 |  |  |
|  | *Endlicheria melinonii* Benoist | CO.66 | 1/10 |  |  |
|  | *Ocotea* *cinerea* van der Werff | MC.125 | 1/10 |  |  |
|  | *Persea nivea* Mez | Gr.1656 | 1/10 |  |  |
|  | *Rhodostemonodaphne rufovirgata* Madriñán | Pr.Gr.4362 | 1/10 |  |  |
|  | Lauraceae sp. 1 | CO.318 | 1/10 |  |  |
|  | Lauraceae sp. undet | Gr.3179 | 1/10 |  |  |
| *Panaunap* | ***Aiouea longipetiolata*** van der Werff | MC.155 | 2/5 | **2/3** | **3/3** |
|  | *Aiouea guianensis* Aubl. | Gr.3155 | 1/5 |  |  |
|  | Lauraceae sp. 4 | CO.20 | 1/5 |  |  |
|  | Lauraceae sp. 5 | CO.338 | 1/5 |  |  |
| *Sedri kamwi* | ***Licaria martiniana*** (Mez) Kosterm. | CO.231 | 3/7 | **1/4** | **4/4** |
|  | *Aniba parviflora* (Meisn.) Mez | CO.93 | 1/7 |  |  |
|  | *Ocotea* sp.14 | CO.112 | 1/7 |  |  |
|  | *Rhodostemonodaphne leptoclada* Madriñán | CO.343 | 1/7 |  |  |
|  | Lauraceae sp. 2 | CO.158 | 1/7 |  |  |
| *Wen* | ***Ocotea guianensis*** Aubl. | Gr.2068 | 7/8 | **1/2** | **2/2** |
|  | Lauraceae sp. 3 | CO.170 | 1/8 |  |  |
| **Lecythidaceae** |  |  |  |  |  |
| *Avun* | ***Eschweilera coriacea*** (DC.) S.A. Mori | Gr.Pr.1990 | 5/38 | **5/6** | **6/6** |
|  | ***Lecythis persistens*** Sagot | CO.27 | 5/38 |  |  |
|  | *Eschweilera grandiflora* (Aubl.) Sandwith | MC.212 | 4/38 |  |  |
|  | *Eschweilera sagotiana* Miers | CO.254 | 4/38 |  |  |
|  | *Eschweilera apiculata*(Miers) A.C.Sm. | MC.116 | 3/38 |  |  |
|  | *Lecythis holcogyne* (Sandwith) S.A. Mori | CO.148 | 3/38 |  |  |
|  | *Eschweilera chartaceifolia* S.A. Mori | MC.321 | 2/38 |  |  |
|  | *Eschweilera micrantha* (O.Berg) Miers | Gr.3128 | 1/38 |  |  |
|  | *Eschweilera parviflora* (Aubl.) Miers | MC.347 | 1/38 |  |  |
|  | *Eschweilera pedicellata* (Rich.) S.A.Mori | MC.452 | 1/38 |  |  |
|  | *Eschweilera* sp. undet. | MC.29 | 1/38 |  |  |
|  | *Eschweilera* sp. undet. | Gr.3031 | 2/38 |  |  |
|  | *Lecythis corrugata* Poit. | L.824 | 1/38 |  |  |
|  | *Lecythis idatimon* Aubl. | Gr.3141 | 1/38 |  |  |
|  | Lecythidaceae sp. 3 | CO.275 | 1/38 |  |  |
|  | Lecythidaceae sp. 7 | CO.270 | 1/38 |  |  |
|  | Lecythidaceae sp. 8 | CO.63 | 1/38 |  |  |
|  | Lecythidaceae sp. 9 | CO.260 | 1/38 |  |  |
| *Kwatri waxriune* (*seyne*) | ***Lecythis poiteaui*** O. Berg | CO.19 | 6/21 | **3/3** | **3/3** |
|  | *Eschweilera* cf. *simiorum* (Benoist) Eyma | CO.160 | 3/21 |  |  |
|  | *Eschweilera chartaceifolia* S.A. Mori | MC.60 | 2/21 |  |  |
|  | *Eschweilera apiculata*(Miers) A.C.Sm. | CO.346 | 1/21 |  |  |
|  | *Eschweilera* cf. *grandiflora* (Aubl.) Sandwith | CO.101 | 1/21 |  |  |
|  | *Eschweilera* cf. *squamata* S.A. Mori | CO.223 | 1/21 |  |  |
|  | *Eschweilera praealta* (Sprague) Sandwith | MC.48 | 1/21 |  |  |
|  | *Eschweilera simiorum* (Benoist) Eyma | MC.608 | 1/21 |  |  |
|  | *Eschweilera* sp. undet. | MC.29 | 1/21 |  |  |
|  | *Eschweilera* sp. undet. | MC.785 | 1/21 |  |  |
|  | *Lecythis holcogyne* (Sandwith) S.A. Mori | MC.48 | 1/21 |  |  |
|  | *Lecythis persistens* Sagot | CO.354 | 1/21 |  |  |
|  | *Corythophora rimosa* W.A.Rodrigues | Gr.3046 | 1/21 |  |  |
| *Wakukwa adava* | ***Gustavia augusta*** L. | MFP.1365 | 4/8 | **1/1** | **1/1** |
|  | *Lecythis zabucajo* Aubl. | Gr.3078 | 2/8 |  |  |
|  | *Eschweilera sagotiana* Miers | CO.44 | 1/8 |  |  |
|  | *Gustavia hexapetala* (Aubl.) Sm. | Pr.Gr.4346 | 1/8 |  |  |
| **Leguminosae** |  |  |  |  |  |
| *ãjelik* | ***Dicorynia guianensis*** Amshoff | Gr.3057 | 10/10 | **3/3** | **3/3** |
| *Kaybune ã* | ***Zygia racemosa*** (Ducke) Barneby & J.W. Grimes | CO.4 | 3/3 | **1/1** | **1/1** |
| *Miumiu* | ***Inga paraensis*** Ducke | Gr.3175 | 3/13 | **1/3** | **3/3** |
|  | *Inga capitata* Desv. | Gr.JLG.3216 | 2/13 |  |  |
|  | *Inga* sp. 15 | CO.332 | 2/13 |  |  |
|  | *Inga acrocephala* Steud. | MC.14 | 1/13 |  |  |
|  | *Inga huberi* Ducke | MC.99 | 1/13 |  |  |
|  | *Inga marginata* Willd. | Pr.Gr.4328 | 1/13 |  |  |
|  | *Inga* sp. 14 | MC.51 | 1/13 |  |  |
|  | *Inga umbellifera* (Vahl) DC. | MC.751 | 1/13 |  |  |
|  | *Tachigali paraensis* (Huber) Barneby | Gr.3006 | 1/13 |  |  |
| *Sakeg* (*kamwi*) | *Hymenolobium flavum* Kleinhoonte | Gr.3067 | 2/13 | **0/2** | **2/2** |
|  | *Parkia nitida* Miq. | Gr.3030 | 2/13 |  |  |
|  | *Calliandra surinamensis*Benth. | L.777 | 1/13 |  |  |
|  | *Enterolobium schomburgkii*(Benth.) Benth. | Gr.2139 | 1/13 |  |  |
|  | *Enterolobium* sp. undet | MC.309 | 1/13 |  |  |
|  | *Macrolobium huberianum*Ducke | CO.105 | 1/13 |  |  |
|  | *Parkia decussata*Ducke | MC.4 | 1/13 |  |  |
|  | *Parkia pendula*(Willd.) Walp. | MFP.1373 | 1/13 |  |  |
|  | *Parkia velutina*Benoist | Gr.1659 | 1/13 |  |  |
|  | *Hymenolobium* sp. undet | Gr.3024 | 1/13 |  |  |
|  | *Zygia racemosa* (Ducke) Barneby & J.W. Grimes | CO.126 | 1/13 |  |  |
| *ã danõ* | ***Bocoa prouacensis*** Aubl. | Gr.3269 | 5/5 | **0/2** | **2/2** |
| *Wakap* | ***Vouacapoua americana*** Aubl. | Gr.3011 | 16/16 | **4/4** | **2/4** |
| *Wap* | ***Eperua falcata*** Aubl. | CO.52 | 4/12 | **4/4** | **4/4** |
|  | *Dialium guianense* (Aubl.) Sandwith | Gr.1766 | 2/12 |  |  |
|  | *Eperua grandiflora* (Aubl.) Benth. | Gr.3082 | 2/12 |  |  |
|  | *Macrolobium bifolium*(Aubl.) Pers. | Gr.1662 | 2/12 |  |  |
|  | *Eperua rubiginosa*Miq. | L.793 | 1/12 |  |  |
|  | *Eperua* sp. 1 | CO.340 | 1/12 |  |  |
| *Yuhumwi* | ***Pentaclethra macroloba*** (Willd.) Kuntze | Gr.1643 | 3/5 | **1/1** | **1/1** |
|  | *Enterolobium* *oldemanii* Barneby & J.W.Grimes | CO.351 | 1/5 |  |  |
|  | *Stryphnodendron guianense* (Aubl.) Benth. | Pr.Gr.4391 | 1/5 |  |  |
| **Melastomataceae** |  |  |  |  |  |
| *Ahayumna* | ***Mouriri nervosa*** Pilg. | Gr.3153 | 6/8 | **3/5** | **5/5** |
|  | *Mouriri francavillana* Cogn. | L.836 | 2/8 |  |  |
| *Avitkat* | ***Mouriri francavillana*** Cogn. | DD.14 | 3/5 | **1/3** | **3/3** |
|  | *Mouriri crassifolia* Sagot | CO.323 | 2/5 |  |  |
| *Timuvukti* | ***Mouriri sagotiana*** Triana | Gr.1627 | 7/8 | **0/3** | **3/3** |
|  | *Mouriri nervosa* Pilg. | CO.88 | 1/8 |  |  |
| **Moraceae** |  |  |  |  |  |
| *Impitit* *waxriune* | ***Maquira guianensis*** Aubl. | CO.311 | 3/4 | **0/1** | **1/1** |
|  | *Brosimum rubescens* Taub. | CO.301 | 1/4 |  |  |
| *Pairi* | ***Brosimum rubescens*** Taub. | CO.172 | 6/16 | **1/2** | **2/2** |
|  | *Brosimum guianense* (Aubl.) Huber ex Ducke | MC.6 | 3/16 |  |  |
|  | *Trymatococcus oligandrus* (Benoist) Lanj. | Gr.3143 | 3/16 |  |  |
|  | *Helicostylis tomentosa* (Poepp. & Endl.) J.F.Macbr. | Pr.Gr.4270 | 1/16 |  |  |
|  | *Pseudolmedia laevigata* Trécul | MC.482 | 1/16 |  |  |
|  | *Pseudolmedia laevis* (Ruiz & Pav.) J.F.Macbr. | Pr.Gr.4286 | 1/16 |  |  |
|  | *Trymatococcus amazonicus* Poepp. & Endl. | Pr.Gr.4337 | 1/16 |  |  |
| *Pakaad* | ***Bagassa guianensis*** Aubl. | Gr.1655 | 2/2 | **1/1** | **1/1** |
| *Tukwangu* | *Helicostylis tomentosa* (Poepp. & Endl.) J.F.Macbr. | MC.508 | 2/7 | **0/2** | **2/2** |
|  | *Naucleopsis guianensis* (Mildbr.) C.C. Berg | Gr.3148 | 2/7 |  |  |
|  | *Pseudolmedia laevis* (Ruiz & Pav.) J.F.Macbr. | CO.227 | 2/7 |  |  |
|  | *Brosimum* sp. | MC.348 | 1/7 |  |  |
| **Myristicaceae** |  |  |  |  |  |
| *Wahusi* (*waxriune*) | ***Virola michelii*** Heckel | Gr.3036 | 7/20 | **0/3** | **3/3** |
|  | *Iryanthera hostmannii* (Benth.) Warb. | Gr.1754 | 3/20 |  |  |
|  | *Iryanthera sagotiana* (Benth.) Warb. | Pr.Gr.4392 | 3/20 |  |  |
|  | *Virola kwatae* Sabatier | MC.20 | 2/20 |  |  |
|  | *Virola multicostata* Ducke | MC.417 | 2/20 |  |  |
|  | *Virola surinamensis* (Rol. ex Rottb.) Warb. | DD.15 | 2/20 |  |  |
|  | *Virola* sp. 2 | MC.698 | 1/20 |  |  |
| **Myrtaceae** |  |  |  |  |  |
| *Awaw* | ***Myrciaria floribunda*** (H. West ex Willd.) O. Berg | Gr.3002 | 4/9 | **2/3** | **3/3** |
|  | *Myrcia fallax* (Rich.) DC. | MC.773 | 1/9 |  |  |
|  | *Myrcia multiflora* (Lam.) DC. | Gr.3113 | 1/9 |  |  |
|  | aff. Myrciaria sp. 1 | CO.253 | 1/9 |  |  |
|  | Myrtaceae A sp. 5 | CO.262 | 1/9 |  |  |
|  | Myrtaceae A sp. 8 | CO.9 | 1/9 |  |  |
| *Inam etni* | ***Eugenia coffeifolia*** DC. | CO.287 | 6/22 | **4/5** | **5/5** |
|  | *Eugenia patrisii* Vahl | Gr.3091 | 3/22 |  |  |
|  | *Myrcia decorticans* DC. | MC.49 | 3/22 |  |  |
|  | *Eugenia* sp.FG13 (Holst) | MC.77 | 2/22 |  |  |
|  | aff. *Eugenia patens* Poir. | CO.68 | 1/22 |  |  |
|  | *Eugenia* aff. *albicans* (O. Berg) Urb. | CO.349 | 1/22 |  |  |
|  | *Eugenia* cf. *florida* DC. | CO.273 | 1/22 |  |  |
|  | *Eugenia* sp. FG-16 | MC.64 | 1/22 |  |  |
|  | *Myrcia aff. Graciliflora* Sagot | CO.358 | 1/22 |  |  |
|  | *Myrcia platyclada*DC. | MC.657 | 1/22 |  |  |
|  | Myrtaceae FG-2 | MC.690 | 1/22 |  |  |
|  | Myrtaceae sp. 23 | MC.100 | 1/22 |  |  |
| *Kagegut* | ***Myrcia fallax*** (Rich.) DC. | Gr.1802 | 2/3 | **1/1** | **1/1** |
|  | *Eugenia pseudopsidium* Jacq. | Gr.3004 | 1/3 |  |  |
| **Ochnaceae** |  |  |  |  |  |
| *Kwatri waxriune* (*duwõ*) | *Lacunaria* cf. *jenmanii* (Oliv.) Ducke | CO.353 | 2/6 | **1/1** | **1/1** |
|  | *Quiina sessilis* Choisy | MC.503 | 2/6 |  |  |
|  | *Quiina obovata* Tul. | MC.492 | 1/6 |  |  |
|  | *Quiina oiapocensis* Pires | MC.718 | 1/6 |  |  |
| **Olacaceae** |  |  |  |  |  |
| *Aneku* | ***Ptychopetalum olacoides*** Benth. | CM.1060 | 5/5 | **0/1** | **1/1** |
| *Yawu* | ***Minquartia guianensis*** Aubl. | Gr.3007 | 5/5 | **5/5** | **0/5** |
| **Rubiaceae** |  |  |  |  |  |
| *ã wakaha* | *Ferdinandusa paraensis* Ducke | MC.480 | 1/2 | **0/1** | **1/1** |
|  | *Ferdinandusa* sp. 1 | CO.299 | 1/2 |  |  |
| *Kinuwup* | ***Duroia eriopila*** L.f. | Gr.1760 | 2/4 | **1/2** | **1/2** |
|  | *Duroia* *aquatica* (Aubl.) Bremek. | MC.472 | 1/4 |  |  |
|  | Rubiaceae sp. 2 | CO.161 | 1/4 |  |  |
| **Sapindaceae** |  |  |  |  |  |
| *Mbagwi* | ***Cupania scrobiculata*** Rich. | CO.147 | 4/9 | **0/2** | **2/2** |
|  | *Matayba* sp. undet | MC.235 | 2/9 |  |  |
|  | *Cupania rubiginosa* (Poir.) Radlk. | MC.128 | 1/9 |  |  |
|  | Sapindaceae sp. undet | MC.334 | 2/9 |  |  |
| *Tuu* | *Talisia carinata* Radlk. | Gr.1585 | 1/5 | **0/3** | **3/3** |
|  | *Talisia megaphylla* Sagot | Gr.1753 | 1/5 |  |  |
|  | *Talisia mollis* Kunth ex Cambess. | CO.185 | 1/5 |  |  |
|  | *Talisia* sp. undet | MC.588 | 1/5 |  |  |
|  | *Toulicia elliptica* Radlk. | L.788 | 1/5 |  |  |
| **Sapotaceae** |  |  |  |  |  |
| *Balata* (*duwõ*) | ***Manilkara huberi*** (Ducke) A. Chev. | Gr.3035 | 3/4 | **2/2** | **2/2** |
| *Kuyaw kamwi* | ***Pouteria decorticans*** T.D. Penn. | MC.83 | 23/36 | **6/6** | **6/6** |
|  | *Pouteria gongrijpii* Eyma | Pr.Gr.4277 | 7/36 |  |  |
|  | *Pouteria filipes* Eyma | Gr.3177 | 2/36 |  |  |
|  | *Pouteria singularis* T.D.Penn. | MC.160 | 2/36 |  |  |
|  | *Pouteria reticulata* (Engl.) Eyma | CO.155 | 1/36 |  |  |
|  | Sapotaceae sp. 1 | CO.157 | 1/36 |  |  |
| *Tukuyuy kamwi* | ***Pouteria jariensis*** Pires & T.D.Penn. | Gr.3183 | 2/4 | **2/2** | **2/2** |
|  | *Pouteria aubrevillei* Bernardi | CO.272 | 1/4 |  |  |
|  | *Pouteria gongrijpii* Eyma | CO.85 | 1/4 |  |  |
| *Uu kamwi* | ***Micropholis cayennensis*** T.D.Penn. | Gr.3059 | 5/56 | **8/8** | **8/8** |
|  | ***Pouteria gongrijpii*** Eyma | Pr.Gr.4293 | 5/56 |  |  |
|  | ***Pouteria torta*** (Mart.) Radlk. | CO.106 | 5/56 |  |  |
|  | *Pouteria rodriguesiana* Pires & T.D.Penn. | Gr.3130 | 4/56 |  |  |
|  | *Pouteria guianensis* Aubl. | CO.252 | 4/56 |  |  |
|  | *Pouteria aubrevillei* Bernardi | MC.108 | 3/56 |  |  |
|  | *Pouteria decorticans* T.D. Penn. | CO.33 | 3/56 |  |  |
|  | *Pouteria macrocarpa* (Mart.) D.Dietr. | CO.111 | 3/56 |  |  |
|  | *Pouteria singularis* T.D.Penn. | CO.290 | 3/56 |  |  |
|  | *Micropholis guyanensis* (A.DC.) Pierre | Gr.1749 | 2/56 |  |  |
|  | *Pouteria hispida* Eyma | CO.154 | 2/56 |  |  |
|  | *Pouteria reticulata* (Engl.) Eyma | MC.699 | 2/56 |  |  |
|  | *Chrysophyllum cuneifolium*(Rudge) A.DC. | CO.127 | 1/56 |  |  |
|  | *Chrysophyllum prieurii*A.DC. | CO.219 | 1/56 |  |  |
|  | *Chrysophyllum* sp.7 | CO.79 | 1/56 |  |  |
|  | *Micropholis venulosa* (Mart. & Eichler ex Miq.) Pierre | MC.484 | 1/56 |  |  |
|  | *Pouteria* aff. *Flavilatex* T.D. Penn. | CO.303 | 1/56 |  |  |
|  | *Pouteria coriacea* (Pierre) Pierre | MC.549 | 1/56 |  |  |
|  | *Pouteria grandis* Eyma | CO.23 | 1/56 |  |  |
|  | *Pouteria laevigata* (Mart.) Radlk. | CO.61 | 1/56 |  |  |
|  | *Pouteria macrophylla* (Lam.) Eyma | Gr.3092 | 1/56 |  |  |
|  | *Pouteria petiolata* T.D.Penn. | MC.527 | 1/56 |  |  |
|  | *Pouteria platyphylla* (A.C.Sm.) Baehni | MC.411 | 1/56 |  |  |
|  | *Pouteria* sp. 31 | MC.711 | 1/56 |  |  |
|  | *Pradosia cochlearia* (Lecomte) T.D.Penn. | Gr.3061 | 1/56 |  |  |
|  | *Sarcaulus brasiliensis* (A.DC.) Eyma | CO.288 | 1/56 |  |  |
|  | Sapotaceae sp. 2 | CO.103 | 1/56 |  |  |
| **Siparunaceae** |  |  |  |  |  |
| *Avakni avak* | ***Siparuna pachyantha*** A.C. Sm. | Gr.3172 | 2/2 | **1/1** | **1/1** |
| *Yahiwemna* | ***Siparuna cristata*** (Poepp. & Endl.) A.DC. | CO.284 | 3/6 | **0/1** | **1/1** |
|  | *Siparuna guianensis* Aubl. | Gr.1850 | 2/6 |  |  |
|  | *Siparuna poeppigii* (Tul.) A. DC. | DG.10315 | 1/6 |  |  |
